# Resolution of inflammation during rheumatoid arthritis

**DOI:** 10.3389/fcell.2025.1556359

**Published:** 2025-03-26

**Authors:** Xiaoou Ye, Dan Ren, Qingyuan Chen, Jiquan Shen, Bo Wang, Songquan Wu, Hongliang Zhang

**Affiliations:** ^1^ Center of Disease Immunity and Intervention, College of Medicine, Lishui University, Lishui, China; ^2^ Department of Orthopedic Surgery, The First Affiliated Hospital of Lishui University, Lishui, China; ^3^ Wenzhou Medical University Affiliated Lishui Hospital, Lishui, China

**Keywords:** rheumatoid arthritis, inflammation resolution, macrophages, fibroblast-like synoviocytes, cytokines

## Abstract

Rheumatoid arthritis (RA) is a chronic autoimmune disease that causes synovial joint inflammation as well as bone destruction and erosion, typically characterized by joint pain, swelling, and stiffness, with complications and persistent pain after remission posing a significant health burden for RA patients. The etiology of RA has not yet been fully elucidated, but a large number of studies have shown that the initiation of inflammation in RA is closely related to T-cell activation, the production of a variety of pro-inflammatory cytokines, macrophage M1/M2 imbalance, homeostatic imbalance of the intestinal flora, fibroblast-like synoviocytes (FLSs) and synovial tissue macrophages (STMs) in the synovial lumen of joints that exhibit an aggressive phenotype. While the resolution of RA is less discussed, therefore, we provided a systematic review of the relevant remission mechanisms including blocking T cell activation, regulating macrophage polarization status, modulating the signaling pathway of FLSs, modulating the subpopulation of STMs, and inhibiting the relevant inflammatory factors, as well as the probable causes of persistent arthritis pain after the remission of RA and its pain management methods. Achieving resolution in RA is crucial for improving the quality of life and long-term prognosis of patients. Thus, understanding these mechanisms provide novel potential for further drug development and treatment of RA.

## 1 Introduction

RA is a chronic autoimmune disease that causes inflammation of the synovial joints and bone destruction and erosion, typically characterized by joint pain, swelling and stiffness (Brown et al., 2024; Conrad et al., 2023; McInnes and Schett, 2011; Mueller et al., 2021). In addition, RA also causes damage to other tissues and organs such as the heart, kidneys, lungs, digestive system, eyes, skin, and nervous system (Giles, 2019; Wu et al., 2022). It is worth noting that about 40% of RA patients suffer from complications, and the incidence of serious complications is 8.3%, with cardiovascular disease, interstitial lung disease, osteoporosis, and metabolic syndrome predominating (Taylor et al., 2021). RA is widespread worldwide, with a prevalence of 0.5%–2%, with a higher prevalence in women, smokers, and those with a family history (Myasoedova et al., 2020).

At present, the etiology of RA has not yet been fully elucidated, and its heterogeneity precludes a single, unified description of pathogenesis. It has been suggested that a combination of genetic factors [e.g., variation at human leukocyte antigen (HLA) loci], environmental exposures (e.g., smoking), and the microbiota contribute to the development of RA (Brown et al., 2024; Smolen et al., 2018). Among them, HLA alleles, especially the HLA-DR1 and HLA-DR4 loci, play an important role in the development of RA by mechanisms that may be related to the formation of citrullinated antigens and immune tolerance of CD4^+^ T cells (Wysocki et al., 2020; Nagafuchi et al., 2022). Sustained antigenic stimulation provided by environmental factors leads to an immune response by inducing the activation of peptidylarginine deiminase (PAD) to produce citrullinated antigens, which stimulate autoantibody production and form immune complexes (Klareskog et al., 2005; Malmström et al., 2016). In addition, pathogenic microorganisms can influence the homeostatic imbalance of the intestinal flora, thereby affecting the immune system and damaging the mesenteric mucosal barrier, promoting joint damage in RA (Maeda et al., 2016). But the most noteworthy immune processes occur in the synovial membrane and synovial fluid (Nygaard and Firestein, 2020; Hu et al., 2019). During the process of RA, joint damage or transient infections can activate the vascular system and allow the entry of autoantibodies. They form immune complexes with joint tissue antigens, activate stromal cells, resident macrophages, mast cells, and osteoclasts, release vasoactive mediators and chemokines, recruit leukocytes, and exacerbate inflammation (Malmström et al., 2016; Smolen et al., 2016). For example, STMs release cytokines such as tumor necrosis factor α (TNF-α), interleukin-1 (IL-1), and interleukin-6 (IL-6) (Ding et al., 2023; McInnes and Schett, 2017). FLSs are stimulated by these cytokines to release inflammatory mediators, chemokines, etc., which promote the influx and proliferation of immune cells. And then, inflammatory factors and FLSs stimulate osteoclast activity, leading to the progression of bone erosion (Yoshitomi, 2019). In addition, activated FLSs produce large amounts of matrix metalloproteinase (MMPs) and receptor activator of nuclear factor-κB ligand (RANKL), which further accelerate the degradation of intra-articular cartilage and bone tissue, leading to further bone erosion development (Ospelt et al., 2008).

Previous treatments often used glucocorticoids or nonsteroidal anti-inflammatory drugs (NSAIDs) (e.g., Ibuprofen and Celecoxib), which, at the cost of significant toxicity, slowed down joint damage and disability at best. In contrast, contemporary therapeutic strategies aim to induce remission of RA and prevent damage before it occurs (Schett et al., 2021; Prasad et al., 2023). In this review, we will focus on summarizing the mechanisms of dissipation and remission of inflammation during the period of RA. We will also discuss the possible causes of persistent arthritis pain after RA remission and its pain management approaches.

## 2 Involvement of immune cells in the remission mechanism of RA

### 2.1 Macrophages

Macrophages are pivotal effector cells that play a pivotal role in the pathogenesis of RA through their capacity to differentiate into various functional phenotypes (Wang et al., 2019). An imbalance in immune homeostasis is created when there are more pro-inflammatory M1-type macrophages than anti-inflammatory M2-type macrophages (Cutolo et al., 2022; Stein et al., 1992; Pan et al., 2021). This imbalance leads to an exacerbation of joint inflammation. Reprogramming of pro-inflammatory macrophages toward anti-inflammatory phenotypes is therefore considered to be an effective way to target the M1/M2 imbalance. Modulating macrophage polarization in order to correct the M1/M2 imbalance represents a promising therapeutic strategy (Zheng et al., 2024a). Metabolically engineered exosomes enable targeted reprogramming of macrophages. When systemically administered into mice with collagen-induced arthritis (CIA), these exosomes effectively accumulated in the inflamed joints, induce M2 polarization, substantially suppresses inflammation in joints and provides strong chondroprotection and osteoprotection (You et al., 2021). Encapsulated a plasmid DNA encoding the anti-inflammatory cytokine interleukin-10 (IL-10pDNA) and the chemotherapeutic drug betamethasone sodium phosphate (BSP) into biomimetic vector M2 exosomes (M2 Exo) derived from M2-type macrophages from M2-type macrophages to form the M2 Exo/pDNA/BSP co-delivery system. This system can promote macrophage polarization from M1 to M2 through the synergistic effect of IL-10 pDNA and BSP in reducing the secretion of pro-inflammatory cytokines (IL-1β, TNF-α) and increasing the expression of IL-10 cytokine (Li et al., 2022). Meanwhile, M2 macrophages-derived extracellular vesicle (EVs) could also convert activated M1 macrophages into M2 macrophages through *in situ* macrophage reprogramming and producing a distinct protein expression pattern characteristic of anti-inflammatory M2 macrophages. After administration of M2-EVs into the joint of a CIA mouse model, the STMs polarization was significantly shifted from M1 to M2 phenotype in decreased joint swelling, arthritic index score and synovial inflammation, with corresponding reductions in bone erosion and articular cartilage damage (Kim et al., 2022a). In addition to this, nanoparticle-based drug delivery is a feasible and common strategy for the treatment of RA. Folic acid-modified silver nanoparticles (FA-AgNPs) are bioactive nanoparticles. After entering the cells, FA-AgNPs dissolved and released Ag^+^ in response to intracellular glutathione, which exerts a series of anti-inflammatory functions, induces apoptosis in M1 macrophages, scavenges reactive oxygen species (ROS), and promotes polarization of M2 macrophages, targeted RA therapy via simultaneous M1 macrophage apoptosis and M1/M2 macrophages reprogramming (Yang et al., 2021). Celastrol-loaded enzyme-responsive nanoparticles dually target osteoclasts and inflammatory macrophages derived from patients with RA, leading to increased apoptosis of these cells. And In an adjuvant-induced arthritis (AIA) rat model, it effectively reduced the number of osteoclasts and inflammatory macrophages in these joints, relieving inflammation and repairing bone erosion (Deng et al., 2021). Nuclear factor-kappa B (NF-κB), Janus kinase (JAK) 1/2 and Extracellular signal-regulated kinase (ERK1)/Notch1 are also potential targets that could selectively suppress proinflammatory macrophages (Zerrouk et al., 2024). JAK inhibitors are potential RA drugs. Phosphodiesterase 3B (PDE3B) is a member of the phosphohydrolase family, which plays a role in numerous signal transduction pathways. The intravenous administration of liposomes loaded with PDE3B siRNA was observed to promote the macrophage anti-inflammatory program, reduce the inflammatory response and synoviocyte infiltration, and attenuate bone and cartilage erosion in CIA mice. A mechanistic study revealed that the depletion of PDE3B increased cAMP levels, which enhanced the PKA-CREB-C/EBPβ pathway and resulted in the transcription of anti-inflammatory program-related genes (Chen et al., 2024). Polyphyllin I (PPI), a principal constituent of Paris polyphyllin, exhibits a selective inhibitory effect on a range of tumor cells. PPI has been shown to suppress the NF-κB-mediated production of pro-inflammatory effectors in activated macrophages, thereby demonstrating effective amelioration of synovial inflammation in the ankle joint of CIA mice (Wang et al., 2018). In addition, by down-regulating hypoxia-associated peptidyl arginine deiminase 4 (PADI4) expression, the number of M1 macrophages was reduced, and the degree of joint swelling and destruction was effectively alleviated in CIA rats, implying that the inflammatory environment can be eased by decreasing PADI4 expression and improving the hypoxic environment (Cheng et al., 2021). In addition, many other mechanisms are involved in regulating the phenotypic transition from M1 to M2 (Feng et al., 2024; Ross et al., 2021).

### 2.2 T cells

T cells are deeply involved in the pathogenesis of RA, especially CD4^+^ T cells drive inflammation in RA joints (Takeshita et al., 2019; Farh et al., 2014; Hu et al., 2011). Th17 can promote synovitis by producing a variety of pro-inflammatory cytokines, and notably, regulatory T cells (Tregs) can suppress inflammatory responses and maintain immune tolerance (Inamo et al., 2021; Wu et al., 2020). Therefore, this paragraph explores the alleviation of RA symptoms by blocking T-cell activation, inhibiting CD4^+^ T-cell pyroptosis, reducing Th17 cell differentiation, and modulating the balance of Th17/Treg and T follicular helper (Tfh)/T follicular regulatory cells (Tfr) (Wu et al., 2020; Yang et al., 2024; Jiang et al., 2023; Zhang et al., 2020).

RA patients have more pyroptotic CD4^+^ T cells in the blood and synovium compared to healthy individuals. Knockdown or pharmacological inhibition of arachidonate 5-lipoxygenase (ALOX5) was found to inhibit CD4^+^ T cell pyroptosis and ameliorate symptoms in two rodent models of RA. The underlying mechanism involves an increase in the activity of ALOX5 in RA CD4^+^ T cells was found to enhance the production of the Leukotriene A4 (LTA4) derivative Leukotriene B4 (LTB4), which stimulated Ca^2+^ influx through Orai protein 3 (ORAI3) channels. This resulted in the activation of NLRP3 inflammasomes and pyroptosis (Cai et al., 2024). Interfering with T cell activation Rho GTPase activating protein (TAGAP) significantly reduced the levels of RhoA and NLRP3 thereby decreasing the differentiation of Th17 cells, alleviating the inflammation and swelling of ankle joints and slowing down the progression of RA in CIA rats (Sun et al., 2023). α-Mangostin (MG) also inhibits Th17 cell differentiation through activation of choline anti-inflammatory pathway (CAP), reduces secretion of TNF-α and IL-1β under inflammatory conditions, and improves the peripheral immune milieu and alleviates inflammatory responses in CIA rats (Yin et al., 2020). Studies have found that Tregs are functionally defective in RA patients (Firestein and McInnes, 2017). Therefore, restoring their function not only controls inflammation, but also restores tolerance in these patients (Jang et al., 2022; Mueller et al., 2021; Byng-Maddick and Ehrenstein, 2015). Interleukin-2 (IL-2) is a pleiotropic cytokine that promote inflammatory response and maintains immune tolerance, and low-dose IL-2 therapy can mediate the progression toward immune tolerance in a variety of autoimmune diseases (Spolski et al., 2018; Rosenzwajg et al., 2015; Hartemann et al., 2013; Malek, 2008). Studies have shown that IL-2 exerts anti-inflammatory effects and maintains immune tolerance by regulating the balance of Th17/Treg and Tfh/Tfr, and is a potential treatment for RA (Wu et al., 2020). Specifically, Tregs highly express the IL-2 receptor alpha chain (CD25), making them highly sensitive to IL-2. Although other T cells also express IL-2 receptors, at low doses, IL-2 has a weaker effect on their proliferation and activation. Clinically relevant drugs that improve arthritis by targeting T cells are available. Abatacept is the only T cell co-stimulation modulator approved thus far for the treatment of moderate-to-severe RA (Schiff, 2015). Abatacept treatment significantly reduced the proportions of Tregs and PD-1^+^ TFh cells and is effective in clinical patients with RA (Aldridge et al., 2022). While abatacept is a potential RA target drug, it may also leading side effects like infection, allergy and tumor. In addition to this, tocilizumab (TCZ), a monoclonal antibody of IL-6R, significantly reduced the number of synovial T cells (Chatzidionysiou et al., 2021). TCZ may also increase the risk of infection, allergy and tumor. The DNA-methylation inhibitors decitabine a potential to ameliorate chronic and acute animal models of RA. by relying on the fact that indoleamine 2,3-dioxygenase (IDO) can generate a regulatory T-cell population that promotes apoptosis of Th1 and Th17 cells (Huang et al., 2021a). While decitabine may also cause myelosuppression, increased risk of infection, gastrointestinal reactions, fatigue and fever. Notably, recent studies have demonstrated sustained drug-free remission using chimeric antigen receptor T cell (CAR-T) therapy for a longstanding case of RA with diffuse large B-cell lymphoma (DLBCL) (Szabo et al., 2024). Besides, there are several clinical trails processed in RA patients under CAR-T treatment. For example, KYV101, an anti-CD19 CAR-T, was developed to against autologous B cells. SBT777101, a CAR-Treg, was also developed to promote Treg proliferation (Table 1). Although these CAR-T treatments possess huge potential for RA patients, they may also leading side effects (e.g., Cytokine Release Syndrome, Immune Effector Cell-Associated Neurotoxicity Syndrome, B-cell Aplasia and Hypogammaglobulinemia, Tumor Lysis Syndrome and off-taget). Moreover, certain T-cell subsets employ their anti-inflammatory attributes to facilitate joint repair. Consequently, the artificial reprogramming of T-cell subsets through the modulation of their metabolic state is anticipated to pave the way for the development of novel molecules that could potentially address RA in the future (Mondal et al., 2024) (Table 1).

**TABLE 1:** Potential drug targets for RA treatment.

Drugs	Active ingredients	Stage	Action effects	Approved time/clinical ID
Abatacept	Fusion protein of CTLA-4 and Fc	FDA approved	Anti-CD80 and CD86 to blocks T cells	2006
Otelixizumab	Monoclonal antibody target CD3	Phase 1	Anti-CD3 to inhibit T cells	NCT01077531
Low dose IL-2	IL-2	Phase 2	Pomote Treg proliferation	NCT01988506
SBT777101	CAR-Treg	Phase 1	Pomote Treg proliferation	NCT06201416
KYV101	Anti-CD19 CAR-T	Phase 1&2	Against autologous B cells	NCT06475495
Rituximab	Monoclonal anti-CD20 antibody	FDA approved	Anti-CD20 to inhibit B cells	2022
VAY736	Monoclonal anti-Baff-R antibody	Phase 1	Target Baff-R to inhibit B cells	NCT03574545
Atacicept	Fusion protein of TACI receptor	Phase 2	Target BLyS and April to inhibit B cells	NCT00430495
Inebilizumab	Anti-CD19 cytolytic monoclonal antibody	Phase 2	B-cell depletion	NCT06570798
GS-0272	Small molecule chemical inhibitor of BTLA	Phase 1	Target BTLA to inhibit T and B cells	NCT06031415
Imvotamab	Monoclonal antibody of CD20 and CD3	Phase 1	Inhibit T and B cells	NCT06087406
VIB4920	CD40L antagonist and Tn3 fusion protein	Phase 2	Inhibit T‐B cell interaction	NCT05306353
IMB-101	Monoclonal antibody of OX40L and TNF-α	Phase 1	Inhibit T-B interaction and TNF function	NCT06181786
KD6005	Fusion protein of TNFR2, BCMA and FC	Phase 1	Inhibit TNF-α, BAFF and April	NCT06213259
Secukinumab	Fully human monoclonal anti-IL-17A	Phase 3	Blocking the actions of IL-17A	NCT01377012
BMS-582949	Small Molecule target MAPK	Phase 2	Inhibit MAPK activation	NCT00605735
R-2487	Probiotic	Phase 1	Improve gut microbiota	NCT05961592
Nicotinamide	Compound functioning as a component of the coenzyme NAD	Phase 2	Anti-inflammatory and antioxidant	NCT06640309
Tofacitinib	Small Molecule inhibitor of JAK1 and JAK3	FDA approved	Inhibit JAK1 and JAK3	2012
Baricitinib	Small Molecule inhibitor of JAK1 and JAK2	FDA approved	Inhibit JAK1 and JAK2	2018
Upadacitinib	Small Molecule inhibitor of JAK1	FDA approved	Inhibit JAK1	2019
Anakinra	Recombinant human IL-1Ra	FDA approved	Binds to the IL-1R and inhibit IL-1	2001
Tocilizumab	Monoclonal antibody of IL-6R	FDA approved	IL-6 inhibiting	2010
Sarilumab				2017
Etanercept	Fusion protein of TNFR and Fc	FDA approved	Inhibit TNF-α	1998
Adalimumab	Monoclonal anti-TNF-α antibody			2002
Golimumab				2013
Infliximab				2019
Certolizumab pegol				2019
MK-8457	Small Molecule inhibitor of Syk	Phase 2	Inhibit Syk	NCT01651936
IMVT-1402	Monoclonal anti FcRn antibody	Phase 2	Inhibit FcRn	NCT06754462
TJ003234	Monoclonal anti GM-CSF antibody	Phase 1	Inhibit GM-CSF	NCT04457856

**FIGURE 1:**
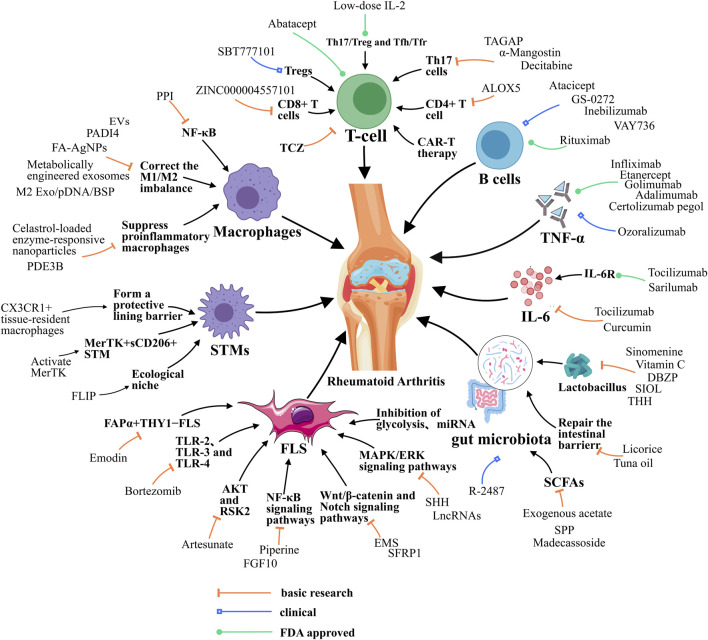
Resolution of Inflammation during RA. Resolution of RA requires the balance between M1/M2 macrophage, the function of T helper cells, STMs and FLSs, and the function of cytokines like IL-6. Besides, gut microbiota also contributes to RA resolution. EVs, extracellular vesicle; FA-AgNPs, Folic acid-modified silver nanoparticles; NF-κB, Nuclear factor-kappa B; JAK, Janus kinase; ERK1, Extracellular signal-regulated kinase; MAPK/ERK, Mitogen-Activated Protein Kinase/Extracellular Signal-Regulated Kinase; PDE3B, Phosphodiesterase 3B; PPI, Polyphyllin I; PADI4, peptidyl arginine deiminase 4; ALOX5, arachidonate 5-lipoxygenase; TAGAP, T cell activation Rho GTPase activating protein; TCZ, tocilizumab; CAR-T, chimeric antigen receptor T cell; SHH, Sonic Hedgehog; EMS, Er Miao San; SFRP1, Secreted frizzled-related protein 1; microRNA, miRNA; lncRNAs, long noncoding RNAs; FLIP, FLICE-like inhibitory protein; DBZP, Polysaccharides from Dianbaizhu; SIOL, Saussurea involucrata oral liquid; THH, Tripterygium hypoglaucum Hutch; SPP, Sporidiobolus pararoseus polysaccharide; FGF10, fibroblast growth factor 10; TLR-2, Toll-like receptor 2; STMs, synovial macrophages; M2 Exo, M2 exosomes; BSP, betamethasone sodium phosphate; IL-10pDNA, Encapsulated a plasmid DNA encoding the anti-inflammatory cytokine interleukin-10; RSK2, Ribosomal S6 Kinase 2.

Besides CD4^+^ T cells, CD8^+^ T cells also drive inflammation in RA. CD8^+^ T cells are notably abundant in synovium and make more interferon (IFN)-γ and nearly as much TNF as CD4^+^ T cells (Jonsson et al., 2022). The majority of these synovium CD8^+^ T cells are granzyme K (GzmK) and granzyme B (GzmB) double positive and acts as cytokines producers to drive inflammation (Jonsson et al., 2022). In addition, CD8^+^ T cells sourced GzmK, a tryptase-like protease, can cleaving fibroblasts produced complement proteins to activate the complement cascade and drive RA inflammation (Donado et al., 2025). GzmK is distinct from GzmB, and currently lack corresponding inhibitors due to limited research. GzmB is produced by NK cells, Cytotoxic T lymphocytes (CTLs) and macrophages during RA and validated for RA treatment. ZINC000004557101, a natural compound, is a potential drug targeting GzmB for treating RA (Wang et al., 2022). B cells are also central to the pathogenesis of RA, contributing to autoantibody production, inflammation, and immune dysregulation, and B cells depletion with anti-CD20 antibody significantly improve RA symptoms (Roll et al., 2006) (Figure 1).

## 3 Involvement of synovial stromal cells in the mechanism of RA remission

### 3.1 Fibroblast-like synoviocytes

In RA, FLSs become a key driver of synovial inflammation and joint damage (Nygaard and Firestein, 2020; Tsaltskan and Firestein, 2022; Bartok and Firestein, 2009). FLSs play a pro-inflammatory role through the production of MMPs and cytokines, osteoclast activation, and immune cell recruitment (Diarra et al., 2007; Yamamura et al., 2001; Reparon-Schuijt et al., 2000). This paragraph therefore summarizes the mechanisms involved in alleviating RA by modulating the signaling pathways, metabolism, and apoptosis of FLSs.

It was found that the Sonic Hedgehog (SHH) signaling pathway may mediate RA-through mitogen-activated protein kinases/extracellular signal-regulated kinases (MAPK/ERK) signaling pathway by regulate proliferation and migration of FLSs, which are involved in the development of RA. In contrast, inhibition of the SHH signaling pathway reduces the proliferation and migration of RA patient-derived FLS (RA-FLS), thereby alleviating RA (Liu et al., 2018; Wang et al., 2014; Elia et al., 2007). Artesunate, an anti-malarial drug, can prevent cartilage and bone destruction in rats with CIA by suppression of PDK1-induced activation of Akt and RSK2 phosphorylation, downregulating the expression of MMP-2 and MMP-9, and inhibiting the migration and invasion of RA-FLS (Ma et al., 2019; Gagliardi et al., 2015). Er Miao San (EMS) may be able to inhibit the activation of the Wnt/β-catenin signaling pathway and inhibit the abnormal activation and angiogenesis of FLSs, thus exerting a protective effect against RA (Chen et al., 2023; Huang et al., 2019). Similarly, Secreted frizzled-related protein 1 (SFRP1) also regulates RA-FLS pyroptosis through Wnt/β-catenin and Notch signaling pathways, suggesting that it could be a promising RA biomarker and therapeutic target (Jiang et al., 2022; Park et al., 2015; Collu et al., 2014; Ando et al., 2003). Aberrant activation of the NF-κB signaling cascade has been identified as the underlying cause of the elevated proliferation and impaired apoptosis of FLSs in RA, which subsequently leads to the onset of inflammation (Li et al., 2013; Roman-Blas and Jimenez, 2006). Piperine was observed to directly reduce the phosphorylation of NF-κB and the expression of NF-κB target genes associated with RA-FLS proliferation, apoptosis inhibition, and inflammation. Additionally, it was noted to increase the expression of apoptosis-related genes and to decrease the protein levels of cytokines and chemokines secreted by FLSs, which resulted in an anti-inflammatory effect (Baito et al., 2023; Pradeep and Kuttan, 2004).

Recent metabolomic studies have demonstrated that metabolic changes, including those in glucose, lipid, and amino acid metabolism, occur prior to the onset of synovitis (Hu et al., 2024; Garcia‐Carbonell et al., 2016). RA-FLS exhibit specific overexpression of glycolytic enzymes, resulting in elevated glycolysis. This elevated glycolysis serves to generate ATP and plays a pivotal role in immune regulation, angiogenesis, and adaptation to hypoxia (Li et al., 2023; Singh et al., 2015). Inhibition of glycolysis may exert a direct effect on synoviocyte-mediated inflammatory functions. Glucose deprivation or incubation of the FLS with glycolytic inhibitors has been observed to impair cytokine secretion and decrease the rate of proliferation and migration of the cells. Furthermore, this has been demonstrated to result in a notable reduction in the severity of arthritis in this mouse model (Garcia‐Carbonell et al., 2016; Ekwall et al., 2011). Furthermore, glycolytic enzymes, such as hexokinase 2 (HK2), are markedly elevated in RA-FLS cells. Consequently, targeting the HK2 enzyme may represent a promising, selective therapeutic target for arthritis, offering a safer alternative to global glycolysis inhibition (Masoumi et al., 2020). Non-coding RNAs have been demonstrated to regulate a number of key biological processes in FLSs, including proliferation, migration, invasion, apoptosis, and inflammatory responses. Additionally, they influence DNA methylation and osteogenic differentiation in FLSs. Given these observations, non-coding RNAs have emerged as a promising avenue for the development of biomarkers for the diagnosis of RA. Local targeting of non-coding RNAs in FLSs represents a promising approach for future therapeutic strategies in RA (Zheng et al., 2024b; Yang et al., 2020; Ye et al., 2017). Several studies have found that microRNA (miRNA) affects the biological properties of RA-FLS. Overexpression of miRNA in RA-FLS significantly inhibited RA-FLS proliferation and promoted apoptosis (Yiou et al., 2023; Hu et al., 2022; Lin et al., 2020). Notably, it has been found that silencing long noncoding RNAs (lncRNAs) nuclear paraspeckle assembly transcript 1 (NEAT1) promotes microRNA-129 and microRNA-204 inhibition of the MAPK/ERK signaling pathway and attenuates FLS synovitis in RA (Chen et al., 2021).

In addition to this, it is also important to alleviate the development of RA by down-regulating various cytokines. It was found that Phospholipase C-like 1 (PLCL1) may promote the inflammatory response in RA-FLS by regulating NLRP3 inflammatory vesicles. Silencing PLCL1 with the NLRP3 inhibitor INF39 resulted in the downregulation of cytokine levels of IL-6, IL-1β, and CXCL8, and therefore, INF39 could counteract the release of inflammatory cytokines caused by overexpression of PLCL1 (Luo et al., 2022; Shi et al., 2021). Bortezomib, a proteasome inhibitor, showed significant inhibitory and proapoptotic activity in splenocytes and FLSs in a rat model of AIA, altered the inflammatory cytokine pattern by down-regulating the expression of Toll-like receptor 2 (TLR-2), TLR-3, and TLR-4 in FLSs and reduced the invasiveness of FLSs from rats with AIA (Ospelt et al., 2008; Yannaki et al., 2010; Seibl et al., 2003). Meanwhile, 8-shogaol, a potent molecule, has been shown to have significant inhibitory effects against TNF-α, IL-1β, and IL-17 mediated inflammation and migration in RA-FLS and 3D synovial culture systems. These effects have been observed in the context of RA, where 8-shogaol has been demonstrated to reverse pathologies of the inflamed synovium by targeting TAK1 (Jo et al., 2022; Courties et al., 2010). Furthermore, fibroblast growth factor (FGF) lining FLSs is highly activated in patients with relapse RA. FGF10 knockdown by small interfering RNA in FLSs significantly reduced the expression of receptor activator of NF-κB ligand. Targeted tissue-specific inhibition of FGF10/FGFR1 may therefore provide new opportunities to treat patients with relapse RA (Meng et al., 2023; Tan et al., 2018). Besides, there are FAPα^+^THY1^−^ fibroblasts selectively mediate bone and cartilage damage with little effect on inflammation, and FAPα^+^ THY1^+^ fibroblasts resulted in a more severe and persistent inflammatory arthritis, with minimal effect on bone and cartilage in RA (Croft et al., 2019). Emodin promote the secretion of STMs exosomes, which inhibit the secretion of pro-inflammatory factors by FAPα^+^THY1^+^ FLSs and promote the secretion of anti-inflammatory factors by FAPα^+^THY1^+^ FLSs, thereby inhibiting FAPα^+^THY1^−^FLS proliferation and migration inhibiting RA damage (Cheng and Rong, 2024).

### 3.2 Synovial tissue macrophages

Different subpopulations of STMs exist in the human synovium, and different STMs subpopulations have different homeostatic and regulatory inflammatory functions, and STM have a key role in restoring joint homeostasis (Kurowska-Stolarska and Alivernini, 2017; Alivernini et al., 2017; De Rycke et al., 2005; Koch et al., 1991). It has been found that a subpopulation of STMs, MerTK^+^TREM2^+^ and MerTK^+^LYVE1^+^, have a unique mitigating transcriptome signature and are enriched for negative regulators of inflammation. These STMs induce synovial fibroblasts to undergo repair *in vitro* and promote the abrogation of inflammatory lipid-mediated disorders. Thus, therapeutic enhancement of the functions of MerTK^+^CD206^+^ STM clusters by activation of MerTK with agonists, could facilitate restoration of synovial homeostasis (Huang et al., 2021b; Boutet et al., 2021; Alivernini et al., 2020; A-Gonzalez et al., 2017). At the same time, STMs form an internal immune barrier at the synovial lining and physically isolate the joint, and these barrier-forming macrophages exhibit features typical of epithelial cells, with epithelioid CX3CR1^+^ lining macrophages limiting the inflammatory response by providing tight junction-mediated shielding for intra-articular structures (Kurowska-Stolarska and Alivernini, 2022; Jaitin et al., 2019; Culemann et al., 2019). It has also been found that the synovial tissue macrophage ecological niche plays a key role in maintaining joint homeostasis and suppressing chronic inflammation, and that FLICE-like inhibitory protein (FLIP) is a risk locus for RA. When FLIP is absent, monocytes are regulated to shift toward pro-inflammatory STMs (Huang et al., 2021b; Huang et al., 2015; Huang et al., 2010). Recent studies have all demonstrated important heterogeneity in macrophage phenotype and function, but the specific mechanisms by which STMs subsets that might amplify the inflammatory response or promote the restoration of tissue homeostasis are unclear (You et al., 2021; Knab et al., 2022; Hannemann et al., 2021; Udalova et al., 2016).

## 4 Involvement of cytokines in the mechanism of RA remission

### 4.1 Tumor necrosis factor

Tumor necrosis factor (TNF) is a pleiotropic cytokine involved in many aspects of immune regulation, and anti-TNF biotherapies are considered a breakthrough in the treatment of RA (Davignon et al., 2018; Kalliolias and Ivashkiv, 2015; Feldmann and Maini, 2010). There have been many studies in the past confirming the clinical effectiveness of RA treatments targeting TNF-α (Guo et al., 2018; Mateen et al., 2016; Bjarnadottir et al., 2014; Geiler et al., 2011). TNF inhibitors, such as etanercept, infliximab, adalimumab, golimumab, and certolizumab, have been found to significantly improve treatment outcomes in RA (Jang et al., 2022; Ma and Xu, 2013). However, these conventional anti-TNF-α antibodies are somewhat immunogenicity and form resistant antibodies when used (Ishiwatari-Ogata et al., 2022; Rubbert-Roth et al., 2019). Currently, ozoralizumab, as a new generation antibody, has demonstrated low immunogenicity in the animal models used (Ishiwatari-Ogata et al., 2022), offering the possibility of early improvement of clinical symptoms, optimization of drug bioavailability, enhancement of tissue penetration and reduction of side effects. Ozoralizumab is therefore an ideal candidate for the treatment of RA patients not only at the initial stage of RA onset, but also during secondary failure of anti-TNF-α therapy (Ishiwatari-Ogata et al., 2022; Tsumoto and Takeuchi, 2024; Takeuchi et al., 2022; Keam, 2022) (Table 1).

### 4.2 IL-6

IL-6 is a typical cytokine with pleiotropic and redundant functions (Pandolfi et al., 2020; Kang et al., 2020). Activation of the IL-6 pathway induces inhibitory molecules as well as the presence of sIL-6R and gp130 forms in the blood to regulate its signaling. Both overproduction of IL-6 and dysregulation of the IL-6 signaling pathway can contribute to the onset and progression of RA (Lee, 2018; Hirano et al., 1985). TCZ is an IL-6 inhibitor commonly used in the treatment of RA (Ogata et al., 2019; Ogata et al., 2012). Furthermore, it was shown that curcumin has been found to significantly reduce H3ac levels in the IL-6 promoter as well as IL-6 mRNA expression in RA synovial fibroblasts (RASFs) (Makuch et al., 2021; Wada et al., 2014). Studies have found that simultaneous inhibition of TNF and IL-6 improves efficacy. The combination of anti-TNF and anti-IL-6 antibodies in a mouse model of CIA resulted in sustained long-term remission, histological improvement, and effects on bone remodeling pathways (Wada et al., 2014; Phillips, 2023). Notably, the IL-6 signaling pathway attenuates TNF-α production by plasmacytoid dendritic cells (pDCs) in RA and reduces the inflammatory potential of synovial fibroblasts in RA patients. The study reveals an anti-inflammatory mechanism that can limit pDC-derived TNF-α secretion via IL-6 (Papadaki et al., 2022; Alculumbre et al., 2019; Takagi et al., 2011). In summary, IL-6 exhibits a dual role in RA, acting as both a pro-inflammatory and anti-inflammatory mediator. Therefore, the role of IL-6 in RA requires further investigation to better understand its mechanisms and implications.

## 5 Involvement of gut microbiota in the mechanism of RA remission

An increasing number of studies have reported that the gut microbiota plays an important role in relieving RA through multiple pathways, therefore, this segment investigates whether modulation of the gut-joint axis, gut microbiota, metabolites of intestinal flora, and the intestinal barrier have an effect on relieving RA. Has been hypothesized that the disease begins in the mucosal sites and then transitions to involve the synovial joints, affecting RA through the gut-joint axis (Lin et al., 2023; Zaiss et al., 2021; Xu et al., 2020). Licorice (GC) can repair the intestinal barrier, improve the relative abundance of gut microbiota, adjusts the gut-joint axis to manage immunological imbalance in RA (Yang et al., 2024). The use of vitamin C to interfere with the gut-joint axis to improve gut microbiota imbalance and inhibit the levels of pro-inflammatory cytokines IL-6 and TNF-α associated with RA can effectively alleviate arthritis symptoms in mice (Zhang et al., 2024). Many studies have also shown that drugs can also provide relief from RA by targeting the gut microbiota. Sinomenine (SIN) can alleviate the symptoms of CIA by targeting *Lactobacillus* and microbial tryptophan metabolites to activate the aryl hydrocarbon receptor (AhR) and modulate the Th17/Treg balance (Jiang et al., 2023; Rosser et al., 2020). Polysaccharides from Dianbaizhu (DBZP) alleviate RA by altering the abundance of specific bacteria such as *Lactobacillus* and *Bacteroides* and a number of metabolites in a process that includes affecting the digestion and metabolism of carbohydrates and protein, altering sex hormones levels, and regulating intestinal immune function (Dong et al., 2024). Meanwhile, Saussurea involucrata oral liquid (SIOL) may play a therapeutic role in RA by improving the tricarboxylic acid (TCA) cycle through modulating the relative abundance of *Lactobacillus*, Romboutsia, *Bacteroides* and Alloprevotella (Cong et al., 2022). Tuna oil (TO) regulates gut microbiota and repairs the damaged intestinal epithelial barrier, thereby relieved arthritis severity and joint bone erosion, and ameliorated systemic inflammation (Lu et al., 2021). Tripterygium hypoglaucum Hutch (THH) extract significantly restored the dysbiosis of regulates gut C57BL/6 mice with AIA, with an increase in Bifidobacterium, Akkermansia, and *Lactobacillus*, and a decrease in Butyricimonas, Parabacteroides, and Anaeroplasma significantly alleviated swollen ankle, joint cavity exudation, and articular cartilage destruction in AIA mice (Zheng et al., 2023; Hu et al., 2023). Notably, prevotella histicola from human gut microbiota suppressed the development of arthritis,but the exact mechanism of action is unclear. All of these studies validate the integral role of gut flora in RA remission (Maeda and Takeda, 2017). Short-chain fatty acids (SCFAs) are metabolites from gut microbes involved in the host’s inflammatory response and immunity (Yao et al., 2022). The production of SCFAs (especially acetic acid, propionic acid and butyric acid) indeed increased significantly by the intervention of Sporidiobolus pararoseus polysaccharide (SPP). These SCFAs play an important role in contributing to the reduction of the inflammatory response and alleviation of the symptoms of RA, in maintaining the intestinal barrier function and in regulating immune homeostasis (Liao et al., 2024; Hu et al., 2021). Butyrate is used as a key intermediate linking the regulates gut to RA. Decreased butyrate alters the immune function and intestinal permeability of the intestinal mucosa, leading to a compromised intestinal barrier, where bacteria and their metabolites can enter the bloodstream and reach the distant target tissues of the host, resulting in local inflammation and aggravating arthritis (Cao et al., 2024; He et al., 2022). It has been reported that madecassoside can expansion of the richness of butyrate-producing bacteria-up-regulation of intestinal butyrate level-induction of Treg cell differentiation and IL-10 expression thereby improving arthritis symptoms (Qiao et al., 2020). Moreover, exogenous acetate was observed to reverse CIA mice with exacerbated gut microbial disruption, thereby further confirming the crucial role of the effect of gut microbial metabolite acetate on neutrophils *in vivo* in immune regulation (Jin et al., 2023; Balakrishnan et al., 2021).

## 6 Persistent arthritic pain after resolution of joint inflammation

Persistent arthritic pain following the resolution of joint inflammation is a common and challenging issue in RA. Despite effective control of inflammation through disease-modifying antirheumatic drugs (DMARDs) or biologic therapies, a large proportion of RA patients have persistent moderate to high pain in a state of inflammatory remission, which significantly impacts their quality of life (McWilliams et al., 2019). A study has revealed that over half of patients experienced severe pain at the outset of, or during, the transition from one biological DMARD (e.g., Adalimumab, Tocilizumab, Rituximab, Abatacept and Anakinra) or targeted synthetic DMARD (e.g., Tofacitinib, Baricitinib and Upadacitinib) to another (Kim et al., 2022b; Vergne‐Salle et al., 2020). Pain progression is heterogeneous in people with RA. Persistent arthritic pain may be caused by a variety of underlying factors, including systemic cytokine, simple mechanical stimulations, and increase neuronal innervation or sensitize peripheral nociceptors at the joint site, but the exact mechanisms are unknown (Kim et al., 2022b; Lim, 2022; Lee et al., 2014). Specifically, chronic inflammation in RA can lead to long-term changes in the central nervous system (CNS), resulting in heightened pain sensitivity (hyperalgesia) and pain from non-painful stimuli (allodynia) (Sağ et al., 2020; Lee et al., 2011). Inflammatory mediators such as cytokines, growth factors and chemokines and immune cells can cause direct damage to peripheral nerves, leading to neuropathic pain (Asbury and Fields, 1984; McWilliams and Walsh, 2017). Even after inflammation resolves, structural damage (e.g., cartilage loss, bone erosion, and joint deformity) can persist, causing mechanical pain (McWilliams et al., 2012). Residual inflammation may persist in joints or surrounding tissues, even when clinical markers (e.g., CRP, ESR) and imaging appear normal (McWilliams and Walsh, 2017). Chronic pain in RA is often associated with psychological comorbidities such as depression, anxiety, and catastrophizing, which can amplify pain perception and disability (McWilliams et al., 2012; Odegård et al., 2007).

Interestingly a few patients displayed superior pain-reducing ability with Janus kinase (Jak) inhibitors and baricitinib monotherapy (Kim et al., 2022b; Lim, 2022). It has also been found that neuroinflammation within the dorsal root ganglion (DRG) may upregulate FcγRI (a key immune receptor) signaling after regression of joint inflammation in a mouse model of collagen antibody-induced arthritis (CAIA), and that knocking down the expression of Fcgr1 in the DRG produces a similar analgesic effect and reduces persistent pain (Liu et al., 2022). The persistence of pain and discomfort during the remission period represents a significant challenge. To enhance the alleviation of patients’ suffering and improve their quality of life, it is imperative to continue investigating the specific mechanisms of remission (Kim et al., 2022b).

## 7 Conclusion

RA causes severe joint pain, swelling, and stiffness with many types of complications and persistent pain after remission poses a significant health burden to the patient. Previously, glucocorticosteroids or NSAIDs were commonly used, which at best slowed joint damage and disability at the cost of significant toxicity. In contrast, contemporary therapeutic strategies aim to induce remission of RA and prevent damage before it occurs. Therefore, this paper summarizes and discusses the relevant inflammatory mechanisms of remission in RA, and concludes that remission of RA can be achieved by blocking T-cell activation, modulating macrophage polarization status, modulating FLS signaling pathways, modulating STMs subpopulations, and inhibiting relevant inflammatory factors. An in-depth understanding of the complex mechanisms of disease remission can help develop new therapies that more precisely target disease-causing molecules. On the other hand, the presence of persistent arthritic pain and discomfort after RA remission is still a problem, so further research on the specific mechanisms of remission can also help to better alleviate patients suffering and improve their quality of life.

Besides these above-mentioned targets and drugs, there are classical and supportive RA drugs. Methotrexate (MTX) is a cornerstone drug in the treatment of RA, primarily functioning through multiple mechanisms: inhibiting the production of pro-inflammatory cytokines, modulating the Th1/Th2 balance, and suppressing the proliferation of immune cells like T and B cells. Curcumin and quercetin are two natural compounds that have been studied for their potential benefits in managing RA, while they are not a replacement for conventional RA treatments (e.g., DMARDs). Vitamin supplementation can play a supportive role in RA management by addressing nutrient deficiencies, reducing inflammation, and protecting joint and bone health. Key vitamins like D, C, E, B6, B9, and K have shown promise in improving RA outcomes. However, supplementation should be tailored to individual needs and used in conjunction with conventional RA therapies under medical guidance. Probiotics represent a promising complementary therapy for RA, with potential benefits in immune modulation, inflammation reduction, and gut health improvement. Classical and supportive RA drugs are more affordable due to their relative low price and higher medical insurance cover, while biological DMARDs are expensive and less insurance covered. To get a better management of RA, a personalized combination of these drugs is recommended.

Although there are already many potential targets and available drugs for current RA treatment. Novel targets and treatment strategies are still required to meet the low response and huge side effects under current treatment. Here are some possible future research directions for RA treatment: precision medicine and personalized therapy with specific biomarkers and genetic variations and/or epigenetic modifications, development of novel targets and mechanisms of RA, new drug formats (bispecific antibodies, improve targeting and reduce side effects), and most of all combination therapy of kinds targets and drugs.
